# Complete Chloroplast Genome of *Alternanthera sessilis* and Comparative Analysis with Its Congeneric Invasive Weed *Alternanthera philoxeroides*

**DOI:** 10.3390/genes15050544

**Published:** 2024-04-25

**Authors:** Yuanxin Wang, Xueying Zhao, Qianhui Chen, Jun Yang, Jun Hu, Dong Jia, Ruiyan Ma

**Affiliations:** 1 College of Plant Protection, Shanxi Agricultural University, Taigu 030801, China; wangyuanx1992@163.com (Y.W.); xyzhao1103@163.com (X.Z.); chenqianhui@163.com (Q.C.); yangjuncau@163.com (J.Y.); hujun.yx@163.com (J.H.); 2 Ministerial and Provincial Co-Innovation Centre for Endemic Crops Production with High-Quality and Effciency in Loess Plateau, Taigu 030801, China; 3 State Key Laboratory of Sustainable Dryland Agriculture (in Preparation), Shanxi Agricultural University, Taiyuan 030031, China

**Keywords:** *Alternanthera sessilis*, *Alternanthera philoxeroides*, chloroplast genome, invasive plants, phylogenetic analysis

## Abstract

*Alternanthera sessilis* is considered the closest relative to the invasive weed *Alternanthera philoxeroides* in China, making it an important native species for studying the invasive mechanisms and adaptations of *A. philoxeroides*. Chloroplasts play a crucial role in a plant’s environmental adaptation, with their genomes being pivotal in the evolution and adaptation of both invasive and related species. However, the chloroplast genome of *A. sessilis* has remained unknown until now. In this study, we sequenced and assembled the complete chloroplast genome of *A. sessilis* using high-throughput sequencing. The *A. sessilis* chloroplast genome is 151,935 base pairs long, comprising two inverted repeat regions, a large single copy region, and a small single copy region. This chloroplast genome contains 128 genes, including 8 rRNA-coding genes, 37 tRNA-coding genes, 4 pseudogenes, and 83 protein-coding genes. When compared to the chloroplast genome of the invasive weed *A. philoxeroides* and other Amaranthaceae species, we observed significant variations in the *ccsA*, *ycf1*, and *ycf2* regions in the *A. sessilis* chloroplast genome. Moreover, two genes, *ccsA* and *accD*, were found to be undergoing rapid evolution due to positive selection pressure. The phylogenetic trees were constructed for the Amaranthaceae family, estimating the time of independent species formation between *A. philoxeroides* and *A. sessilis* to be approximately 3.5186–8.8242 million years ago. These findings provide a foundation for understanding the population variation within invasive species among the *Alternanthera* genus.

## 1. Introduction

*Alternanthera philoxeroides*, commonly known as alligator weed, is a perennial herb within the *Alternanthera* genus of the Amaranthaceae family. It originates from the Paraguay and Parana River basins in South America. Over time, it has expanded its presence across a vast geographical range, spanning from 32 degrees north to 38 degrees south latitude, making it a globally pervasive invasive weed that poses significant threats to both the environment and the economy [1,2,3]. *Alternanthera sessilis*, another perennial herb belonging to the same *Alternanthera* genus as *A. philoxeroides*, is believed to be native to tropical and subtropical regions of Asia, northeastern Australia, and the wetlands of tropical America [3,4,5]. These two species share similarities in morphology, with upright or prostrate growth habits, and the ability of all stem nodes to develop roots. Their flowers are axillary in nature. While *A. sessilis* has sessile inflorescences, *A. philoxeroides* exhibits different characteristics. Their distribution areas overlap within China, with *A. sessilis* primarily found along the wet edges of East China, South China, Central China, and Southwest China. Conversely, *A. philoxeroides* is predominantly distributed in the broader regions to the south of the Yangtze River and gradually extends into sporadic areas in North China. It holds a larger territory and exhibits greater competitive strength compared to *A. sessilis*.

Invasive weeds have a remarkable capacity for rapid adaptation to new environments, making them excellent subjects for studying adaptive changes in plants [6,7,8]. One common approach is to compare the adaptability and invasiveness of alien invasive species with their local relatives. As a native species, *A. sessilis* has been frequently employed in studies of the adaptation and invasive mechanisms of *A. philoxeroides*, yielding valuable insights. In contrast to *A. sessilis*, *A. philoxeroides* demonstrates superior photosynthetic capacity, a faster stem growth rate, a broader temperature tolerance range, enhanced competitive abilities, and a greater capacity for invasion [9]. *A. philoxeroides* holds distinct advantages over *A. sessilis*, whether facing biotic or abiotic stressors [10]. Following exposure to herbivores and nematodes, *A. philoxeroides* displays increased branching, facilitating its expansion and invasion [11]. Its defense responses surpass those of *A. sessilis*, making it more resistant to the intrusion of pathogenic microorganisms [12]. Furthermore, *A. philoxeroides* exhibits a wider environmental adaptability range and more robust phenotypic plasticity. It thrives in various aquatic environments and demonstrates heightened tolerance to waterlogging [13]. Its osmotic adjustment capabilities exceed those of its native congener, *A. sessilis* [14,15]. Additionally, *A. philoxeroides* excels in clonal integration, enabling it to outcompete *A. sessilis* within its ecological niche [16]. These findings shed light on the invasion mechanism employed by *A. philoxeroides* to a considerable extent.

Chloroplasts are cellular organelles responsible for photosynthesis in plants and the provision of energy for growth. They also serve as vital hubs for plant signal integration, actively participating in adaptation to environmental stress [17]. Chloroplasts possess a circular genome consisting of four main sections: a large single copy region (LSC), a small single copy region (SSC), and two identical inverted regions (IRs) separated by two unique single copy regions. Typically, chloroplast genomes (cp genome) have a size ranging from 107 kb to 218 kb, containing approximately 120 to 130 genes. The number and arrangement of chloroplast genes (cp genes) exhibit a high degree of conservation, albeit with occasional insertions, deletions, and rearrangements. These attributes of high conservation and slow evolution of chloroplast genomes offer an effective means of distinguishing groups that are challenging to classify based on morphology [18,19]. Cp genes also prove effective in the identification of invasive plants, such as the combined utilization of *matK* and nucleic ITS [20].

The cp genome plays a crucial role in elucidating the relationships and evolutionary dynamics between invasive species and their congeners [21]. By comparing cp genomes across different regions or among related species, researchers can analyze origins, evolutionary pathways, and spread patterns. For example, this approach has been applied to examine invasive and native individuals of *Jacobaea vulgaris* [22], invasive *Mikania micrantha* and its native species *M. cordata* [23], and *Sonchus asper* and *S. oleraceus* [24]. This method has increasingly become a potent tool for plant molecular systematics, phytogeography, and the investigation of intraspecific polymorphism and interspecific divergence [21,25,26]. With advancements in next-generation sequencing technologies, there has been a growing interest in studying the cp genomes of invasive species within specific regions. Despite the sequencing and reporting of the cp genome of *A. philoxeroides* [27], research on the cp genome sequences of its native congener, *A. sessilis*, and their comparative analysis remains limited.

In this study, the complete cp genome of *A. sessilis* was sequenced and assembled. The analysis of hotspot regions and repeat sequences was carried out in comparison to the cp genome of the invasive weed, *A. philoxeroides*. Furthermore, highly divergent regions between *A. sessilis* and *A. philoxeroides* were identified. Using protein-coding genes, a phylogenetic tree of the Amaranthaceae family was constructed, and the divergence time between *A. sessilis* and *A. philoxeroides* was estimated.

## 2. Materials and Methods

### 2.1. Plants Collection

*Alternanthera sessilis* were gathered from a natural population in Meizhou City, Guangdong, China, and subsequently cultivated within a greenhouse at Shanxi Agriculture University (Taigu, China). Once they had bloomed, their seeds were harvested, dried, and stored in a refrigerator for future cultivation. These seeds were then sown in small black square pots measuring 7 cm × 7 cm and cultured under controlled conditions at a temperature of 25 ± 1 °C with a photoperiod of 16 h of light and 8 h of darkness for six weeks to obtain test plants. Fresh leaves were collected after exposure to more than 6 h of light and were promptly frozen using liquid nitrogen for subsequent DNA extraction.

### 2.2. DNA Extraction and Sequencing

The frozen leaves of *A. sessilis*, as described earlier, were pulverized into a fine powder using liquid nitrogen to facilitate total DNA extraction. Total DNA was extracted utilizing the Plant DNA Isolation Kit (Tiangen, Beijing, China). Subsequently, the total DNA was fragmented through ultrasound treatment. The resulting DNA fragments underwent purification, end repair, and adapter ligation. Following PCR enrichment, the fragments were separated through agarose gel electrophoresis and subsequently employed for constructing a DNA library. This library was subjected to sequencing on an Illumina NovaSeq platform (San Diego, CA, USA), generating paired-end reads with a length of 150 bases (PE150).

### 2.3. Assembly and Annotation of A. sessilis Chloroplast Genome

Raw sequencing data were subjected to filtration using fastp 0.20.0 (https://github.com/OpenGene/fastp, accessed on 4 December 2023), which entailed the removal of adapter sequences and reads with an average quality score falling below Q5 or containing more than five ambiguous bases (N) to obtain high-quality clean reads. To simplify the assembly process, these clean reads were aligned against the chloroplast genome database from Genepioneer Biotechnologies (Nanjing, China) using bowtie2 v2.2.4 (http://bowtiebio.sourceforge.net/bowtie2/index.shtml, accessed on 4 December 2023) to specifically identify sequencing reads corresponding to the chloroplast genome. These selected cp genome sequencing reads were subsequently assembled into contigs using SPAdes v3.10.1 (http://cab.spbu.ru/software/spades/, accessed on 4 December 2023). These contigs were further organized into scaffolds using SSPACE v2.0 (https://www.baseclear.com/services/bioinformatics/basetools/sspace-standard/, accessed on 4 December 2023). To obtain a complete, circular cp genome, any gaps between scaffolds were filled using Gapfiller v2.1.1 (https://sourceforge.net/projects/gapfiller/, accessed on 4 December 2023).

Annotation of the CDS within the *A. sessilis* cp genome was carried out using Prodigal v2.6.3 (https://www.github.com/hyattpd/Prodigal, accessed on 4 December 2023). Separately, the prediction of transfer RNA (tRNA) and ribosomal RNA (rRNA) was performed using Aragorn [28] v1.2.38 (http://130.235.244.92/ARAGORN/, accessed on 4 December 2023), tRNAscan-SE [29] (http://trna.ucsc.edu/tRN-Ascan-SE/, accessed on 4 December 2023), and Hmmer v3.1b2 (http://www.hmmer.org/, accessed on 4 December 2023). Additionally, sequence alignment and annotation were conducted based on the gene sequences of related species, and the assembled sequences were subjected to blast v2.6 (https://blast.ncbi.nlm.nih.gov/Blast.cgi, accessed on 4 December 2023) for further annotation. The final annotation result was obtained after manually removing redundancy. The complete cp genome sequence of *A. sessilis* was deposited in the NCBI GenBank with the specific accession number PP239384.

### 2.4. Analysis Data Collection

The cp genome sequences of *A. philoxeroides*, which were used for comparative analysis, were retrieved from the NCBI GenBank (accession number: NC_042798.1). These samples were collected in Jinan, China.

### 2.5. Chloroplast Genome Structure

The analysis of inverted repeat regions (IRs) within the *A. sessilis* cp genome was conducted using GeSeq [30] (https://chlorobox.mpimp-golm.mpg.de/geseq.html, accessed on 18 January 2024). Verification of the large single copy region (LSC), small single copy region (SSC), and IRs was performed using Geneious 10.1 [31]. Visualization of the structure of the *A. sessilis* cp genome was achieved using OGDraw [32] (https://chlorobox.mpimpgolm.mpg.de/OGDraw.html, accessed on 18 January 2024).

### 2.6. Codon Usage Bias Analysis of A. sessilis and A. philoxeroides cp Genomes

Codon usage bias is a widespread phenomenon observed across various species and stages of life. This bias is considered a result of long-term evolution and is influenced by multiple factors, with directional mutation and neutral selection being primary contributors [33,34]. Relative synonymous codon usage (RSCU), a commonly employed parameter for studying codon usage bias, represents the ratio between actual and expected codon occurrences. It aids in the analysis of gene function and evolutionary patterns. An RSCU value of 1 signifies no codon bias, while values greater than 1 indicate a higher occurrence of a codon compared to other synonymous codons, and vice versa. The RSCU values for the cp genomes of *A. sessilis* and *A. philoxeroides* were calculated using the protein-coding genes from these cp genomes. This analysis was performed using codon usage analysis in MEGA 11.0 [35].

### 2.7. Repeat Sequence Analysis

Repetitive sequences, distributed widely throughout the genome, are believed to play a crucial role in gene recombination and rearrangement. The cp genome evolves at a relatively slow pace, with repetitive sequences in non-coding regions exhibiting a higher degree of variability. This characteristic facilitates the characterization of genetic variation at lower taxonomic levels and aids in addressing population genetic inquiries [25,36,37]. The identification of repeats in the cp genome holds great significance for the development of novel molecular markers. REPuter [38] (https://bibiserv.cebitec.unibieleeld.de/reputer/, accessed on 18 January 2024) was employed to detect various types of repeat sequences within the cp genomes of both *A. sessilis* and *A. philoxeroides*. For the analysis of simple tandem repeats, Tandem Repeats Finder [39] (http://tandem.bu.edu/trf/trf.html, accessed on 18 January 2024) was utilized. Simple sequence repeats (SSRs) were identified using MISA [40] (https://webblast.ipk-gatersleben.de/misa/, accessed on 18 January 2024). The SSRs were searched for mononucleotide to hexanucleotide repeat motifs with a minimum repeat number of 10, 5, 4, 3, 3, and 3 for mo, di, tri, tetra, penta, and hexanucleotide repeats, respectively. The compound SSR was identified when the length of a sequence between two SSRs to register was <100 bp.

### 2.8. Analysis of Hotspots and ka/ks and Identification of Highly Divergent Regions

The cp genome sequences of *A. sessilis* and *A. philoxeroides* were aligned using MAFFT 7.037 [41]. Nucleotide polymorphism (Pi) within the cp genomes of these species was analyzed using DnaSP 6.0 [42] to identify regions with high variability, employing a parameter of a 200 bp step size and 600 bp window length. Seventy-one common protein-coding genes were selected to assess the frequency of synonymous and non-synonymous substitution events using DnaSP 6.0, providing insights into evolutionary selection pressures. 

### 2.9. Contraction and Expansion Analysis of IRs Boundaries

The contraction and expansion of IRs have a substantial impact on the size of the cp genome [26,43]. Irscope [44] (https://irscope.shinyapps.io/irapp/, accessed on 18 January 2024) was employed to analyze the contraction and expansion of IRs within the cp genomes of *A. sessilis* and *A. philoxeroides*.

### 2.10. Genome Analysis and Comparison with Other Amaranthaceae Species cp Genomes

Using the annotation of the *A. sessilis* cp genome as a reference, a comprehensive comparison was conducted with the cp genomes of other Amaranthaceae species using mVISTA [45] (http://genome.lbl.gov/vista/mvista/submit.shtml, accessed on 18 January 2024). This analysis aimed to assess the distinctions between their respective cp genomes.

### 2.11. Phylogenetic Analysis and Divergence Time Estimate

To construct the phylogenetic tree, we utilized 59 common protein-coding genes from a total of 28 species. This set included 25 species from the Amaranthaceae family and 3 outgroups consisting of 2 species from the Achatocarpaceae family and *Dianthus caryophyllus*. Sequence alignment was carried out using MAFFT 7.037 [41]. The maximum likelihood (ML) tree was constructed with MEGA 11.0 [35], employing the best model GTR + G + I and 1000 bootstrap replicates. ModelFinder [46] was utilized to determine the best-fit model for constructing Bayesian inference phylogenies. The Bayesian phylogenetic tree was generated using Mybayes 3.2.6 within Phyosuite 1.1.16 [47], employing the best-fit model GTR + F + I + G4 with 2 parallel runs and 2,000,000 generations. The initial 25% of the sampled data was discarded as burn-in.

Divergence times were estimated using the RelTime-ML method with the local molecular clock in MEGA 11 [35]. Calibration points for divergence times were derived from TimeTree [48] (http://timetree.org/, accessed on 23 January 2024), specifically, the divergence time of the *Amaranthus* genus and *Chenopodium* genus (24.5–73.8 MYA) and the *Suaeda* genus and *Salicornia* genus (12.1–39.7 MYA), based on data from 10 and 5 studies, respectively. The time tree was constructed using the maximum likelihood method and the GTR + G + I model.

## 3. Results

### 3.1. Sequencing, Assembly, and Annotation of A. sessilis cp Genome

**Figure 1:**
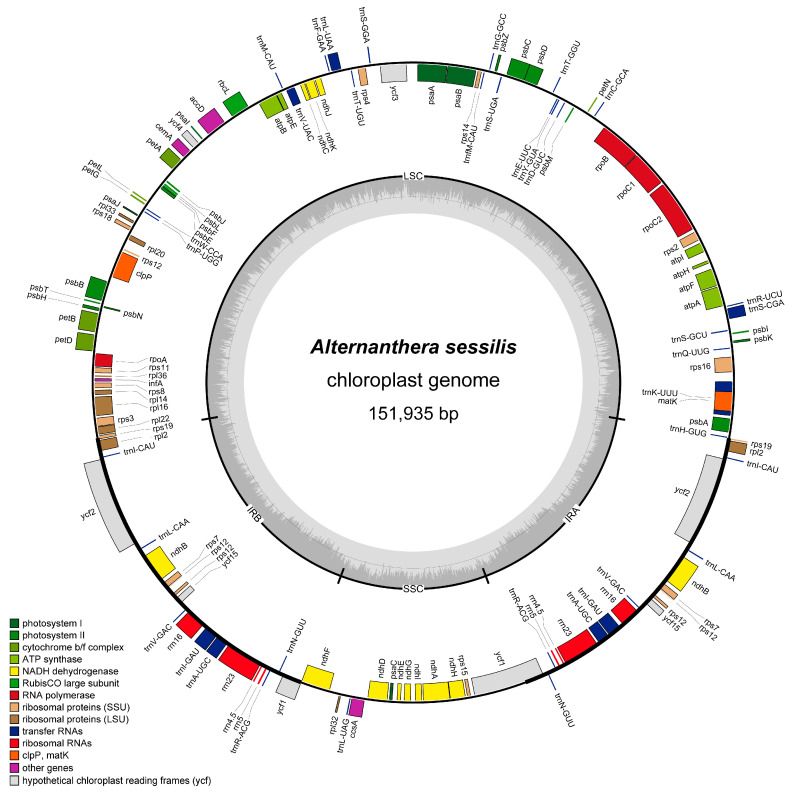
Chloroplast genome map of *Alternanthera sessilis*. Genes coding forward are on the outer circle, while genes coding backward are on the inner circle. The gray circle inside represents the GC content.

High-quality clean reads, totaling 2.54 GB with a Q30 of 92.86%, were obtained and employed for the assembly of the complete cp genome of *A. sessilis*. The *A. sessilis* cp genome is 151,935 bp in length and follows the typical quadripartite structure, comprising two inverted repeat regions (IRs), a large single copy region (LSC), and a small single copy region (SSC). The LSC region spans 84,449 bp, while the SSC region is 17,298 bp long, with a pair of Irs, each covering 25,095 bp (Figure 1). The overall GC content of the *A. sessilis* cp genome is 36.3%, while the GC contents of LSC, SSC, and IRs are 33.3%, 29.8%, and 42.5%, respectively (Table 1). It is noteworthy that IRs exhibit the highest GC content, primarily due to the presence of high-GC-content rRNA genes. A comparison of the *A. sessilis* cp genome with previously reported cp genomes from the Amaranthaceae species revealed similarities with the *A. philoxeroides* cp genome (Table 1).

The *A. sessilis* cp genome encodes a total of 128 genes, including 8 rRNA-coding genes, 37 tRNA-coding genes, 4 pseudogenes, and 83 protein-coding genes. Among the protein-coding genes, 44 are related to photosynthesis, 24 are involved in self-replication, and the remaining 10 have diverse functions (Table 2). In comparison to its close relative, the *A. philoxeroides* cp genome, *A. sessilis* possesses three additional tRNA-coding genes: *trnG-GCC*, *trnS-CGA*, and *trnfM-CAU*. Furthermore, the gene *trnM-CAU* is present in a single copy in the *A. sessilis* cp genome, whereas there are two copies in the *A. philoxeroides* cp genomes. Additionally, the *A. sessilis* cp genome contains two unique protein-coding genes, *rpl22* and *rps15*, absent from the *A. philoxeroides* cp genome. Notably, the *ndhA* genes have different structures in the two species, with *ndhA* in *A. sessilis* having one intron, whereas that in *A. philoxeroides* is an all-exon structure. *A. sessilis* also harbors two specific pseudogenes, *ycf15* and *ycf1*, which contain introns. Within the *A. sessilis* cp genome, eight tRNA-coding genes possess one intron. Among them, two copies of *trnI-GAU* and *trnA-UGC* are located in IRs, while the remaining four tRNA-coding genes are situated in the LSC region. Notably, *trnK-UUU* contains the largest intron, spanning 2538 bp, and encodes the *matK* gene. Eleven protein-coding genes in the *A. sessilis* cp genome contain introns, primarily associated with self-replication and photosynthesis. Nine of these genes possess one intron, predominantly situated in the LSC region, except for *ndhB* in IRs and *ndhA* in SSC. Two protein-coding genes, *clpP*, and *ycf3*, each exhibit two introns (Table 3).

### 3.2. Codon Usage Bias of A. sessilis and A. philoxeroides cp Genomes

**Figure 2:**
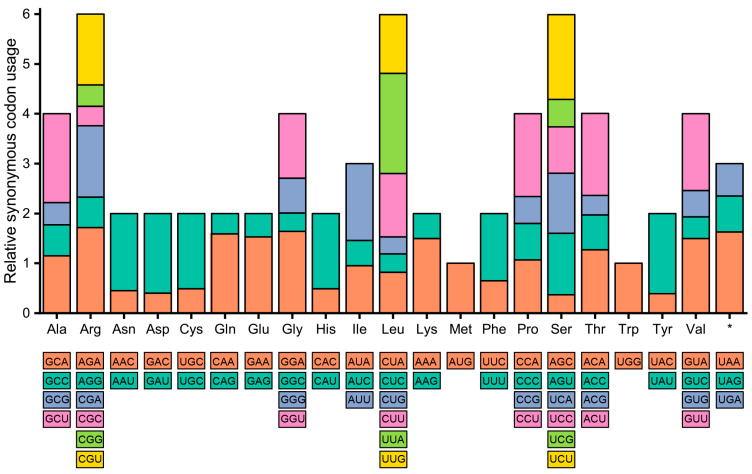
Relative synonymous codon usage in the *A. sessilis* chloroplast genomes. *: Terminator.

The usage of synonymous codons in the cp genomes of *A. sessilis* was assessed using relative synonymous codon usage (RSCU) and compared with that of *A. philoxeroides*. In both genomes, Leu was found to have the highest amino acid frequency, accounting for 10.60% in *A. sessilis* and 10.37% in *A. philoxeroides*, while Cys exhibited the lowest frequency at 1.17% in *A. sessilis* and 1.68% in *A. philoxeroides* (Figure 2). Regarding start codons, in the *A. sessilis* cp genome, ACG was used as the start codon for *psbL*, while GTG was utilized for *rps19*, *ndhD*, and *ycf1*. In the *A. philoxeroides* cp genome, *psbL* and *ndhD* employed ACG as the start codon, while only *rps19* used GTG. The RSCU values for stop codons UAA, UAG, and UGA in the *A. sessilis* cp genome were 1.63, 0.72, and 0.65, respectively. UAA was preferred as the primary stop codon in the *A. sessilis* cp genome. In contrast, a more balanced preference for stop codon usage was observed in the *A. philoxeroides* chloroplast genome, with RSCU values of 1.16 for UAA, 0.95 for UAG, and 0.89 for UGA (Table S1).

### 3.3. SSRs and Long Repeated Sequences

**Figure 3:**
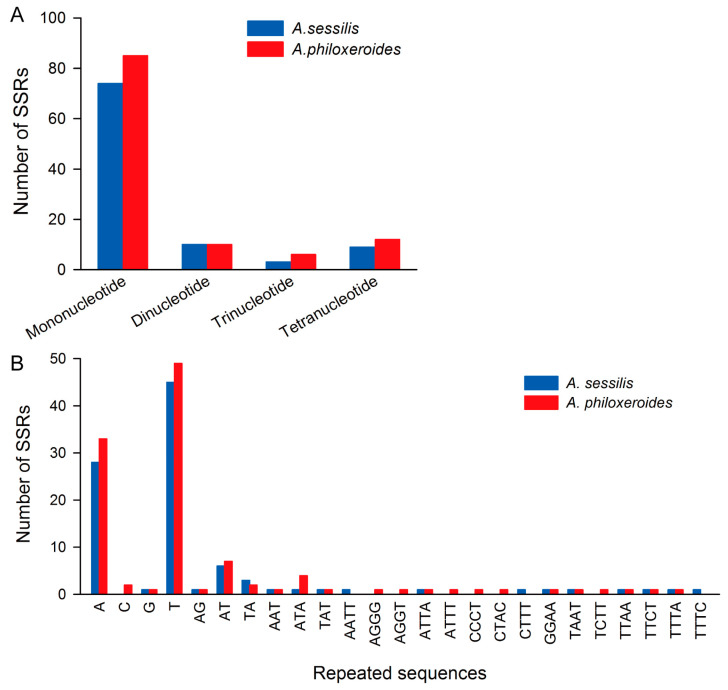
Number of simple sequence repeats in *A. sessilis* and *A. philoxeroides* chloroplast genomes. (**A**) Number of simple sequence repeats of different types in *A. sessilis* and *A. philoxeroides* chloroplast genomes based on the repeating motif length. (**B**) Number of simple sequence repeats with different motif types in *A. sessilis* and *A. philoxeroides* chloroplast genomes.

In the cp genomes of both *A. sessilis* and *A. philoxeroides*, a total of 96 and 113 SSRs of four types were identified, respectively. Generally, *A. philoxeroides* exhibits a higher abundance of SSRs compared to *A. sessilis*. Based on the length of the repeating motifs, the *A. sessilis* cp genome contains 74 single-nucleotide repeat sequences, 10 dinucleotide repeats, 3 trinucleotide repeats, and 9 tetranucleotide repeats. In contrast, *A. philoxeroides* has an equal number of dinucleotide repeats but shows a higher occurrence of single-nucleotide, trinucleotide, and tetranucleotide repeats (Figure 3A). Regarding the type of repeating motif, in the *A. sessilis* cp genome, the most abundant is the T single-nucleotide repeats, followed by A single-nucleotide repeats. These two types of motif repeats account for 60.81% and 37.83%, respectively, out of all single-nucleotide repeats (Figure 3B). A similar distribution pattern was observed in the *A. philoxeroides* cp genome (Figure 3B).

**Figure 4:**
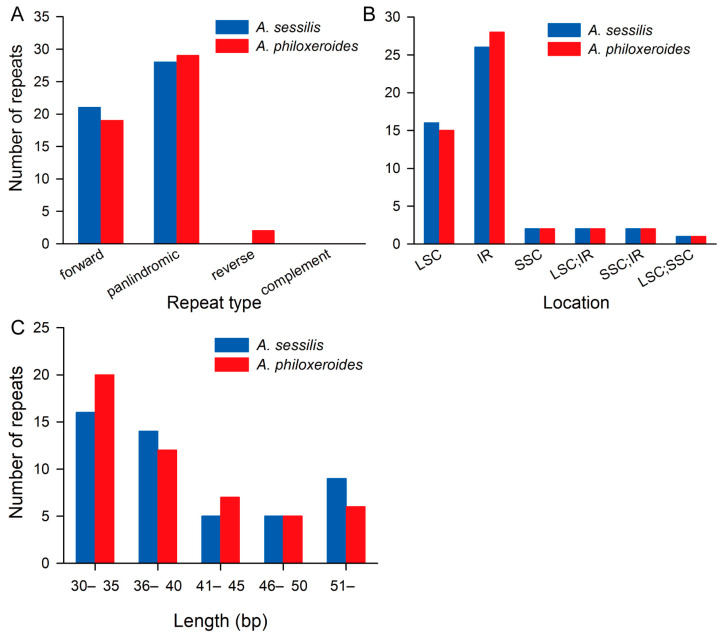
Number of repetitive sequences in *A. sessilis* and *A. philoxeroides* chloroplast genomes. (**A**) Number of repetitive sequences of different types in *A. sessilis* and *A. philoxeroides* chloroplast genomes. (**B**) Number of repetitive sequences in different locations in *A. sessilis* and *A. philoxeroides* chloroplast genomes. (**C**) Number of repetitive sequences of different lengths in *A. sessilis* and *A. philoxeroides* chloroplast genomes.

Forty-nine and fifty repetitive sequences longer than 30 base pairs were identified in the cp genomes of *A. sessilis* and *A. philoxeroides*, respectively. These included 21 forward repeats and 28 palindrome repeats in the *A. sessilis* cp genome and 19 forward repeats, 29 palindrome repeats, and 2 reverse repeats in the *A. philoxeroides* cp genome (Figure 4A). The majority of these large repetitive sequences are located in the LSC and IR regions (Figure 4B). Repeats with lengths ranging from 30 to 40 base pairs account for 61.2% and 64% of the total repetitive sequences in the cp genomes of *A. sessilis* and *A. philoxeroides*, respectively (Figure 4C).

### 3.4. Divergence Hotspots and Ka/Ks

**Figure 5:**
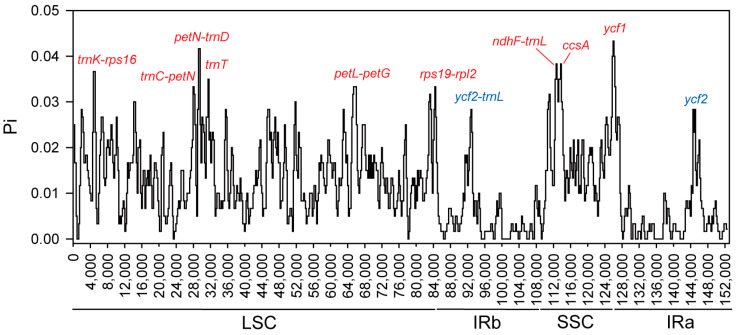
The nucleotide polymorphism for cp genomes of *A. sessilis* and *A. philoxeroides* calculated using DnaSP 6.0 employing parameters of a 200 bp step size and 600 bp window length. Eleven most divergent regions are suggested as mutation hotspots. The name of regions in red indicate these regions are located in LSC region or SSC region, and those in blue indicate the regions are located in IRs.

Despite the structural similarity between the cp genomes of *A. sessilis* and *A. philoxeroides*, notable nucleotide differences exist. Nucleotide polymorphism (Pi) was used as an indicator to measure nucleic acid divergence, ranging from 0 to 0.0433, with an average value of 0.01159. Nine regions with high nucleotide polymorphisms were identified, including *trnK-rps16*, *trnC-petN*, *petN-trnD*, *trnT*, *petL-petG*, *rps19-rpl2*, *ndhF-trnL*, *ccsA*, and *ycf1* (Pi > 0.033). These highly variable regions are primarily distributed in the LSC and SSC regions, while the IR region remains more conserved. Only two regions exhibit higher Pi values: *ycf2-trnL* in the IRa region and ycf2 in the IRb region, with Pi values of 0.02833 (Figure 5).

Synonymous and non-synonymous substitution rates were analyzed for the 73 protein-coding genes shared by the cp genomes of *A. sessilis* and *A. philoxeroides*. The synonymous substitution rate ranged from 0 to 0.0789, with *rps19* exhibiting the highest synonymous substitution rate. The non-synonymous substitution rate ranged from 0 to 0.0294, and *infA* displayed the highest non-synonymous substitution rate (Table S2).

**Figure 6:**
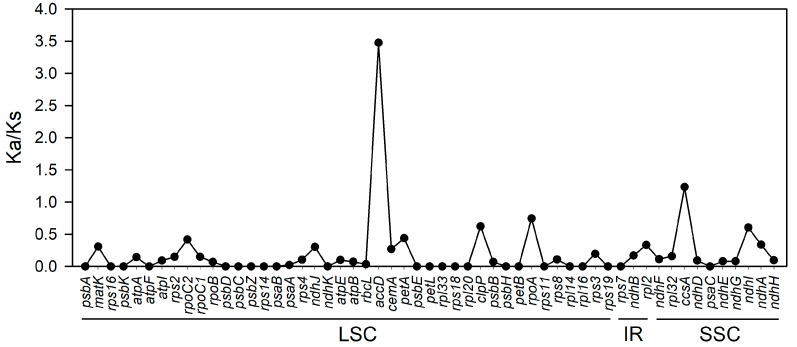
The Ka/Ks ratio of 55 protein-coding genes in *A. sessilis* and *A. philoxeroides* calculated using DnaSP 6.0. Ka/Ks values of *AccD* and *CcsA* were more than 1.

The ratio of synonymous substitution rate to non-synonymous substitution rate (Ka/Ks) was further calculated to assess the selection pressure on 57 protein-encoding genes, with Ka/Ks ratios ranging from 0 to 3.475. Out of these, 55 genes had Ka/Ks ratios below 1, indicating a bias toward purification selection. Notably, 16 genes, including *atpF* and *psbA*, exhibited a Ka/Ks ratio of 0, suggesting that they are under strong purification selection pressure. However, two genes, *ccsA* and *accD*, displayed Ka/Ks ratios above 1, specifically, 1.237 and 3.475, respectively. This suggests that these two genes, especially *accD*, are rapidly evolving under positive selection influence and may play a crucial role in the evolution of the species. For the remaining 16 genes, the Ka/Ks ratio could not be calculated due to Ks = 0 (Figure 6).

### 3.5. Comparison of Chloroplast Genomes in the Amaranthaceae Family

**Figure 7:**
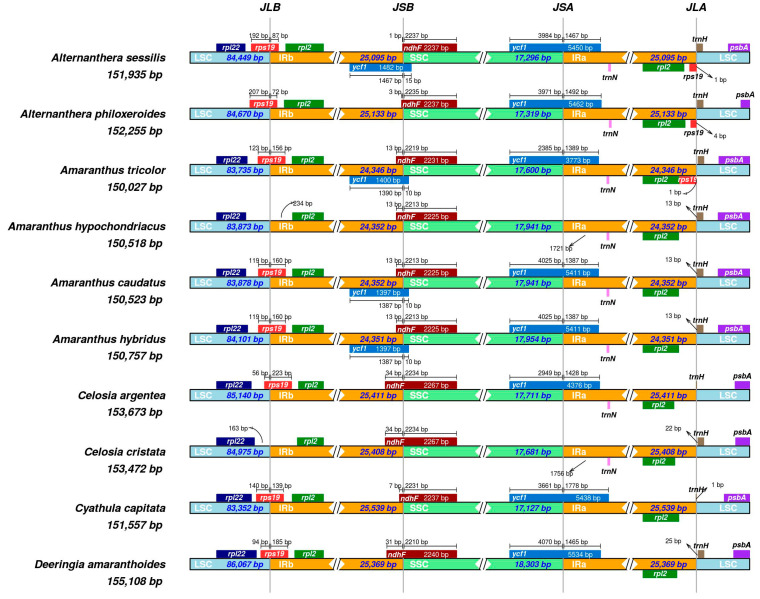
Comparison of the border positions of the LSC, IR, and SSC regions among ten Amaranthaceae species chloroplast genomes. Gene names are indicated in the boxes and their lengths in the corresponding regions are displayed above the boxes.

Among ten species in the Amaranthaceae sensu stricto, their cp genomes exhibit remarkable structural conservation. However, noticeable variations in cp genome size are attributed to the contraction and expansion of the IR boundaries (Figure 7). The IR regions in these cp genomes vary in size, ranging from 24,346 to 25,539 base pairs (bp) (Table 1). Regarding the LSC/IRb boundary, all Amaranthaceae species, except for *A. philoxeroides*, harbor the *rpl22* exclusively within the LSC region, devoid of any cross-boundary coding. With the exception of *Amaranthus hupochondriacus* and *Celosia cristata*, the remaining eight species feature the *rps19* proximal to *rpl22*, extending into the IRb regions with segments spanning 72 to 223 bp, overlapping the LSC/IRb boundary. In *A. sessilis* and *A. philoxeroides*, the *rps19* copy predominantly resides in the LSC region, with minor portions extending into the IRb region (87 bp and 72 bp, respectively). Furthermore, an additional *rps19* copy is found solely within the IRa regions of *A. sessilis*, *A. philoxeroides*, and *A. tricolor*. At the SSC/IRb boundary, a pseudogene of *ycf1* is present in *A. sessilis*, *Amaranthus tricolor*, *Amaranthus caudatus*, and *Amaranthus hybridus*, primarily located in IRbs, extending into the SSC region by 10–15 bp. The premature termination of ORF was observed in the aforementioned pseudogene ycf1 as a result of the contraction and expansion of the IR boundaries. Conversely, *ndhF* genes are primarily situated in the SSC regions, overlapping the SSC/IRb boundary and containing segments approximately 1–34 bp within the IRbs. Moving to the SSC/IRa boundary, *ycf1* genes in these Amaranthaceae species primarily inhabit the SSC regions, extending 1387–1778 bp into the IRa regions. Notably, *A. hypochondriacus* and *C. cristata* lack *ycf1*. Concerning the LSC/IRa boundary, the *trnH* genes in the cp genomes of *C. argentea* and *C. acpitata* are predominantly found in the IRb region, while, in the other species, they are mainly located in the LSC region, with a distance ranging from 1 to 25 bp from the boundary (Figure 7).

**Figure 8:**
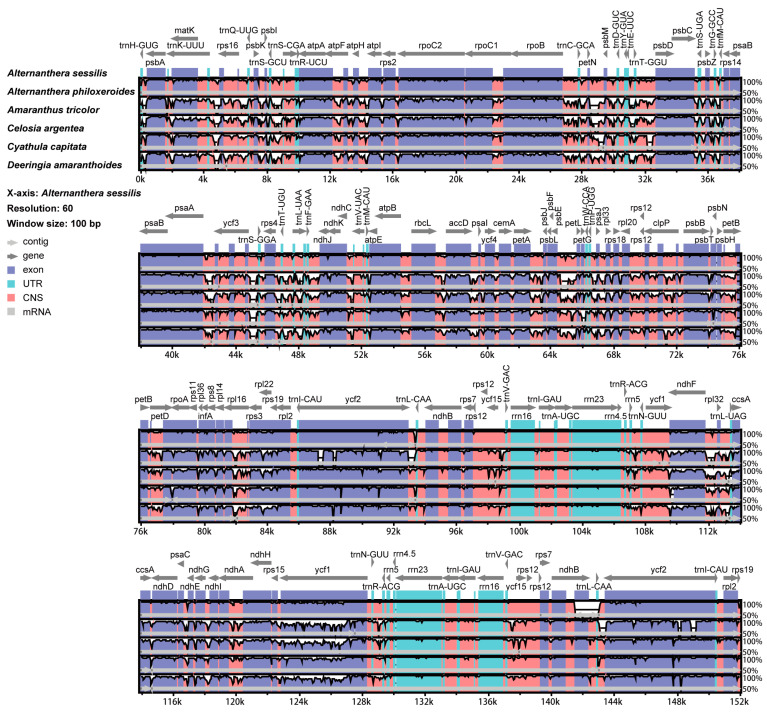
Similarity of chloroplast genome sequences among six Amaranthaceae species from different genera. Sequence identity is portrayed with a cut-off of 50% identity. The Y-scale axis represents the percent identity within 50–100%. Grey arrows indicate genes with their orientation and position. Genome regions are color-coded as purple blocks for the conserved coding genes (exon), light red blocks for the conserved non-coding sequences in intergenic regions (CNS), and aqua blue blocks for UTR. The lines below the alignment indicate the chloroplast genomes. Black-bordered white peaks that are shown in genome regions indicate the divergent regions with sequence variation among six Amaranthaceae species.

Using the *A. sessilis* cp genome sequence as a reference, six known cp genome sequences from five related genera were aligned. The results revealed a high degree of similarity among these sequences, with most regions displaying over 50 percent similarity. Considering the chloroplast structure, relatively high similarity was noted in the IR regions, while lower levels of similarity were observed in the LSC and SSC regions. From the perspective of gene structure, extremely high sequence similarity was found in both exon and UTR regions, except for *ccsA*, *ycf1*, and *ycf2*, which exhibited relatively high diversities. The non-coding region displayed low similarity and significant variation, suggesting its potential as a hotspot for the development of new molecular markers. The intergenic regions, *trnK-rps16*, *petN-trnD*, *petL-petG*, and *ndhF-trnL*, showed large diversity, consistent with the results of single nucleotide polymorphism analysis. Furthermore, significant diversity was observed in the gene introns, such as those within *petD*, *rpl16*, and *ndhA*. In our focused study on the cp genome of *A. sessilis* and *A. philoxeroides*, two regions of low similarity were identified between *ndhB* and *ycf2*, adjacent to *trnL-CAA* (Figure 8).

### 3.6. Phylogenetic Analysis and Estimation of Divergence Time

**Figure 9:**
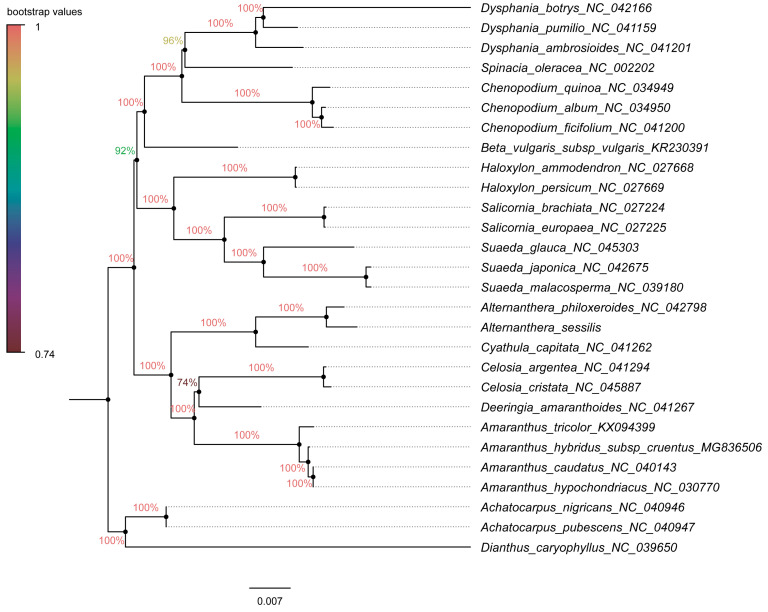
The phylogenetic tree constructed using the maximum likelihood method in MEGA 11.0 with the GTR + G + I model, employing 59 chloroplast gene sequences from 28 species. The chloroplast genome sequences of *Achatocarpus nigricans*, *Achatocarpus pubescens*, and *Dianthus caryophyllus* were used as the outgroup. The numbers on the branches indicate the bootstrap values.

**Figure 10:**
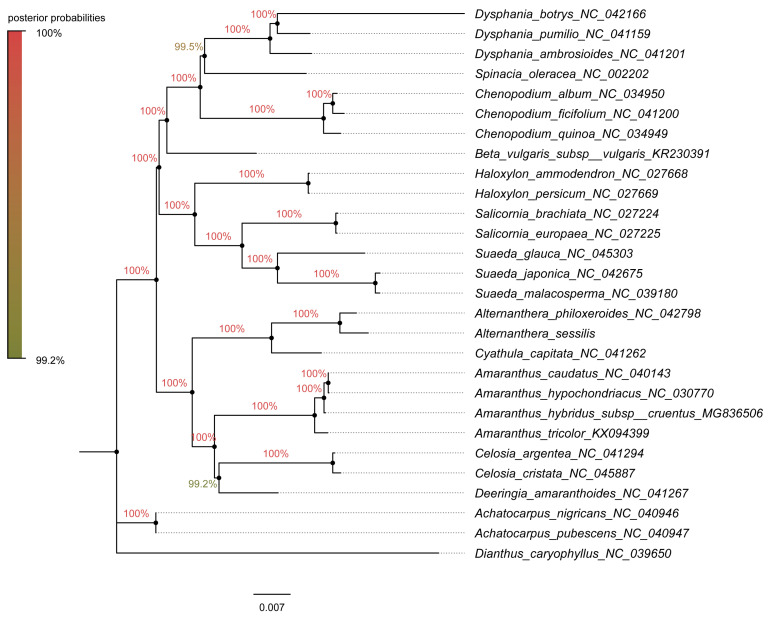
The phylogenetic tree constructed using the Bayesian inference method in Mybayes 3.2.6 under the GTR + I + G + F model, incorporating 59 chloroplast gene sequences from 28 species. The chloroplast genome sequences of *A. nigricans*, *A. pubescens*, and *D. caryophyllus* were employed as the outgroup. The numbers on the branches indicate the posterior probabilities.

For the construction of phylogenetic trees, we utilized fifty-nine chloroplast protein-coding genes from 25 species within Amaranthaceae s.l as the inner group, while *Achatocarpus nigricans*, *Achatocarpus pubescens*, and *Dianthus caryophyllus* were employed as the outgroups. The phylogenetic trees were generated using both the maximum likelihood method and the Bayesian inference method independently. Remarkably, both methods yielded similar tree topologies (Figure 9 and Figure 10). In these trees, *A. sessilis* and *A. philoxeroides* clustered together with the *Cyathula* species, forming one branch, while the genera *Amaranthus*, *Celosia*, and *Deeringias* grouped into another branch. These genera are part of Amaranthaceae s.s and formed a single branch collectively. Species belonging to *Chenopodium*, *Beta*, *Haloxylon*, *Salicornia*, and *Suaeda* genera constituted a distinct branch. Notably, the Chenopodiaceae branch and the Amaranthaceae s.s branch together formed a monophyletic group.

**Figure 11:**
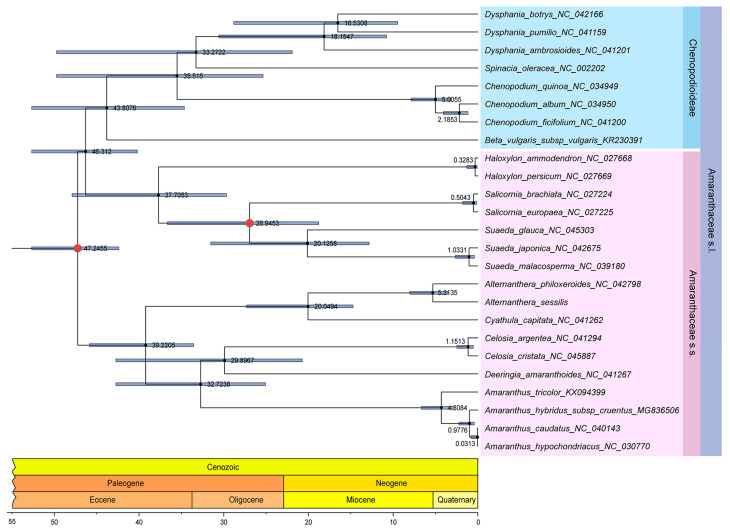
The time tree estimated under the local molecular clock using the RelTime-ML method in MEGA 11.0. The circular representation with full red indicates the divergence times of the *Amaranthus* and *Chenopodium* genus (estimated at 24.5–73.8 MYA) as well as the *Suaeda* and *Salicornia* genus (estimated at 12.1–39.7 MYA), derived from 10 studies and 5 studies, respectively, in TimeTree, used for estimating the divergence time between *A. sessilis* and *A. philoxeroides*.

Additionally, a time tree was constructed using the aforementioned phylogenetic trees and analyzed to estimate divergence times. The results indicated that Amaranthaceae s.s. and Chenopodiaceae likely originated during the Paleogene period, approximately 47.24 MYA. The divergence between the *Alternanthera* genus and the *Cyathula* genus occurred around 20.04 MYA. The independent speciation events for *A. philoxeroides* and *A. sessilis* took place roughly between 3.5186 and 8.8242 MYA (Figure 11).

## 4. Discussion

In this study, we conducted sequencing and annotation of the chloroplast genome of *A. sessilis*. A comparative analysis of cp genomes was performed between relative local species *A. sessilis* and invasive weed *A. philoxeroides*. This study aimed to identify differences in the cp genomes of these two *Alternanthera* species, particularly focusing on hotspot regions and repeat sequences. We also investigated the *accD* and *ccsA* genes, which appear to be evolving rapidly due to positive selection. The divergence time between *A. sessilis* and *A. philoxeroides* was estimated.

Overall, the cp genomes of both *A. sessilis* and *A. philoxeroides* have a size of approximately 150 kb, with GC percentages ranging from 36.3% to 36.8% (Figure 1 and Table 1). These cp genomes exhibit the typical tetrad structure observed in other Amaranthaceae species. Changes in cp genome sizes are typically attributed to variations in the inverted repeat (IR) region, gene loss, and alterations in gene spacer regions [49,50,51]. Our results reveal that various regions within the cp genomes of Amaranthaceae s.s. species have undergone length changes, ranging from a few dozen to thousands of nucleotides. Notably, there is a size variation of approximately 1 kb within the IR regions. To the best of our knowledge, *A. sessilis* and *A. philoxeroides* possess medium-sized IR regions. Among Amaranthaceae s.s. species, the smallest IRs are found in *A. tricolor*, measuring 24,346 bp, while the largest IRs are observed in the cp genome of *C. capitata*, with a length of 25,539 bp.

When comparing the genes and gene numbers in the cp genomes of *A. sessilis* and *A. philoxeroides* with those of other Amaranthaceae species, it is evident that the protein-coding genes in *A. sessilis* and *A. philoxeroides* are similar to those found in other Amaranthaceae species. However, *A. hypochondriacus* and *C. cristata* exhibit a lower number of protein-coding genes. Interestingly, although both *A. sessilis* and *A. philoxeroides* have the same number of protein-coding genes, their genes are different. In the cp genome of *A. sessilis*, genes such as *rpl22* and *rps15* are present, while *rpl23* is absent. Conversely, the cp genome of *A. philoxeroides* (NC_042798), collected from Jinan, exhibits the opposite situation (Table 2). Another *A. philoxeroides* cp genome from Shiyan (MK450441) [27], which contains *rpl22* and *rps15* but lacks *rpl23*, is similar to *A. sessilis*. In general, the loss of plastid genes can be attributed to two main reasons. Firstly, non-essential gene loss results in permanent absence. Secondly, the gene may transfer from the plastid genome to the nuclear genome [52,53]. The loss of *rpl23* in the cp genomes of *A. sessilis* is a common occurrence in Amaranthaceae s.l. Eleven species have demonstrated the loss of *rpl23*, while other species have shown pseudogenization of *rpl23* [54]. It is suggested that both *rpl22* and *rpl23* are essential for leaf development, and their loss can lead to leaf deformities [55]. In Leguminosae, *rpl22* transfers to the nucleus before being lost from the cp genome [56]. This suggests that there may be nuclear transfer of *rpl23*/*rpl22* in the cp genomes of *A. sessilis* and *A. philoxeroides*, resulting in the loss of *rpl23* or *rpl22*, which requires further investigation. Furthermore, the absence of *rps15*, although not essential for plastid translation, can reduce the expression efficiency of chloroplast genes, decrease accumulation levels in photosynthesis complexes, affect ribosomal small subunit specificity, and impact leaf pigments, consequently retarding plant growth and development. Cold stress can exacerbate these negative effects [57]. While the S15 protein encoded by the nucleus can be transported into plastids, it does not fully compensate for the loss of plastid-encoded *rps15* [55]. In the cp genomes of *A. philoxeroides*, the sample from Jinan lacks *rps15*, whereas the sample from Shiyan possesses it, suggesting that invasive weed *A. philoxeroides* in northern regions may have other cold adaptation mechanisms to mitigate the negative effects caused by the loss of *rps15*. All these differences between the two *A. philoxeroides* cp genomes may be attributed to their different geographical locations, highlighting the potential impact of latitude on population variations in alien species like *A. philoxeroides*, which needs more evidence.

The boundaries of the IR region in *A. sessilis* and the other nine Amaranthaceae cp genomes were highly conserved, with minimal variation. This conservation aligns with the typical pattern of IR region boundaries in most angiosperm species [58]. The expansion and contraction of the IR region can lead to the pseudogenization of genes [59]. In our study, we observed that pseudogenes *ψrps19* and *ψycf1* were located at the IR boundary in the chloroplast genome of *A. sessilis* (Figure 7), similar to findings for *Mikania micrantha* and its native congener, *Mikania cordata* [23]. Specifically, *ψrps19* spanned the JLA (LSC/IRb) boundary within Amaranthaceae s.s. Compared to the normal gene, we observed an 83 bp reduction in length at its 3′-end. *ψycf1* crossed the JSB (SSC/IRb) boundary and exhibited base deletions, causing a forward shift in its stop codon position and resulting in a size reduction to 1483 bp. Generally, such genes may be transferred to the nuclear genome [60,61]. However, it is worth noting that additional intact copies of *rps19* and *ycf1* were also present in the chloroplast genome of *A. sessilis*, raising the possibility of nuclear-transferred genes, which requires further investigation. Additionally, we found two copies of pseudogene *ψycf15* in the IR region, both with sizes of 519 bp and incomplete ORFs due to base deletions. In chloroplast genomes, such as those observed in ANA-grade species, monocots, most rosids, etc., *ycf15* genes are fragmented or completely lost due to nuclear gene transfers within the same plant lineages [61,62] or horizontal transfers from different plant lineages [63].

The nucleotide polymorphism in the chloroplast genomes of *A. sessilis* and the related species *A. philoxeroides* was notably high, and significant nucleic acid divergence regions were observed (Figure 5). These divergence regions were primarily situated in single-copy regions and could serve as hotspots for designing DNA barcodes for species identification. They offer potential molecular markers for investigating genetic variation in invasive plants within the Amaranthaceae family. Notably, regions like *trnK-rps16* and *trnC-petN*, characterized by substantial nucleic acid divergence, have been successfully used for identifying traditionally challenging classified species such as oaks [64] and the *Fragaria* genus [19]. Furthermore, these regions can contribute to the phylogenetic analysis of related species, as observed in wild grapes [18]. Additionally, large nucleic acid divergences were also identified in the *petL-petG*, *rps19-rpl2*, *ndhF-trnL*, *ccsA*, and *ycf1* regions. Genes like *petN*, *petL*, and *petG* encode subunits of cytochrome b_6_f, which play a crucial role in photosynthetic electron transport [65], suggesting that these divergences may be related to adaptations to varying light conditions. Furthermore, our study revealed that in the cp genomes of these two *Alternanthera* species, *accD* and *ccsA* are under positive selection pressure (Figure 6). The *accD* encodes the beta subunit of the enzyme acetyl-CoA carboxylase, which supplies malonyl-CoA primarily for fatty acid synthesis [66], playing an important role in leaf development [67]. This positive selection of *accD* was also observed in the chloroplast genome of Firmiana [68]. CcsA encodes cytochrome c biogenesis protein, which mediates the attachment of heme to c-type cytochromes [69]. It has been shown that *ccsA* was under positive selection in the epiphytic orchid [70] and Erigeron [71]. Both of them have been found to contribute to adaption to environmental changes in other species, for example, *Lilium Ledebourii* and the species in Malvaceae subfamilies [72,73]. 

Amaranthaceae s.l. is ranked as the second-largest family within the core Caryophyllales, with a sister group relationship to Achatocarpaceae indicated by molecular phylogenetic evidence [74]. Within this family, Amaranthaceae s.s. forms a clearly monophyletic group. In this study, phylogenetic trees were constructed using 59 chloroplast protein-coding genes across 28 species, employing both the maximum likelihood and Bayesian inference methods. Regardless of the methods and models used, the results were highly consistent, displaying similar topological structures (Figure 9 and Figure 10), which aligned with Yao’s plastid phylogenomic report of Caryophyllales [54]. The phylogenetic relationships within the *Alternanthera* genus were supplemented in our study, a detail not covered in the previous tree. Notably, a strong affinity was observed between the *Alternanthera* genus, comprising *A. sessilis* and *A. philoxeroides*, and the *Cyathula* genus. The incorporation of a broader range of chloroplast protein-coding genes from various genera within Amaranthaceae s.l. in our phylogenetic analysis provided a clearer understanding of the evolutionary and ancestral relationships within this family compared to previous studies. Although previous research had separately examined the phylogenetic relationships within the *Alternanthera* genus in the Americas using combined sequences from the cp genome (*trnL-F* and *rpl16*) and nuclear sequences (ITS sequences) [75], it did not elucidate the relationship between *A. philoxeroides* and *A. sessilis*. The use of complete cp genome sequences to explain their evolution was advantageous for closely related taxa and offered higher resolution [26]. It is important to note, however, that complete cp genome sequences for Amaranthaceae species remain limited. Further research is needed to explore the evolution and ancestral relationships at the subfamily and genus levels within Amaranthaceae.

Subsequently, the divergence time between *A. philoxeroides* and *A. sessilis* was estimated to have occurred approximately 3.5186–8.0242 million years ago, during the transition from the Miocene to the Quaternary period (Figure 11). This timeframe coincided with the wide proliferation of C4 plants and a shift in climate toward an ice age. The *Alternanthera* genus comprises species with various photosynthetic pathways, including C3, C4, and C3-C4 intermediate species [75], with *A. sessilis* being classified as a C3 species [76]. Our results indicated that the rapidly evolving genes in these two *Alternanthera* species were associated with photoadaptation and environmental adaptation, potentially contributing to their invasive capabilities.

## 5. Conclusions

In this study, the complete chloroplast genome of *A. sessilis* was sequenced, assembled, and compared with its relative species. This cp genome is 151,935 base pairs long with a typical quadripartite structure and contains 128 genes, including 8 rRNA-coding genes, 37 tRNA-coding genes, 4 pseudogenes, and 83 protein-coding genes. This chloroplast genome exhibited high conservation in structure and gene contents with other relative species; however, some regions showed signification variations and rapidly evolving genes involved in photosynthesis and environmental adaption were identified. The phylogenetic trees indicated that the *Alternanthera* genus is closely related to the *Cyathula* genus, and *A. philoxeroides* and *A. sessilis* were estimated to have diverged approximately 3.5186–8.8242 million years ago. Our findings lay the foundation for understanding the population variation and environmental adaptability within invasive species among the *Alternanthera* genus.

## Figures and Tables

**Table 1:** Comparison of basic characteristics of chloroplast genomes in Amaranthaceae species.

Species	Genus	GenBank Number	Genome Size (bp)	LSC (bp)	SSC (bp)	IR (bp)	GC (%)	tRNA Gene Number	rRNA Gene Number	Protein-Coding Gene Number	Total Gene Number
*Alternanthera sessilis*	*Alternanthera*	PP239384	151,935	84,449	17,298	25,095	36.3	37	8	83	128
*Alternanthera philoxeroides*	*Alternanthera*	NC_042798	152,255	84,670	17,319	25,133	36.4	35	8	83	126
*Amaranthus tricolor*	*Alternanthera*	KX094399	150,027	83,735	17,600	24,346	36.6	33	8	89	130
*Amaranthus hypochondriacus* cultivar *Plainsman*	*Amaranthus*	NC_030770	150,518	83,873	17,941	24,352	36.6	31	8	73	112
*Amaranthus caudatus*	*Amaranthus*	NC_040143	150,523	83,878	17,941	24,352	36.6	37	8	84	129
*Amaranthus hybridus* subsp. *PI566897*	*Amaranthus*	MG836506	150,757	84,101	17,954	24,351	36.6	37	8	84	129
*Celosia argentea*	*Celosia*	NC_041294	153,673	85,140	17,711	25,411	36.7	37	8	85	130
*Celosia cristata*	*Celosia*	NC_045887	153,472	84,975	17,681	25,408	36.7	36	8	73	117
*Cyathula capitata*	*Cyathula*	NC_041262	151,557	83,350	17,129	25,539	36.4	37	8	84	129
*Deeringia amaranthoides*	*Deeringia*	NC_041267	155,108	86,065	18,305	25,369	36.8	35	8	84	127

**Table 2:** Genes in the chloroplast genome of *Alternanthera sessilis* and *Alternanthera philoxeroides*.

Function of Genes	Category of Genes	Gene Name
Self-replication	Large subunit of ribosome	rpl2 ^a^, rpl14, rpl16 ^b^, rpl20, rpl22(ses), rpl23 ^a^(phi), rpl32, rpl33, rpl36
	DNA-dependent RNA polymerase	rpoA, rpoB, rpoC1 ^b^, rpoC2
	Ribosomal RNA genes	rrn4.5 ^a^, rrn5 ^a^, rrn16 ^a^, rrn23 ^a^
	Small subunit of ribosome	rps2, rps3, rps4, rps7 ^a^, rps8, rps11, rps12 ^abe^, rps14, rps15(ses), rps16 ^b^, rps18, rps19 ^d^(ses), rps19 ^a^(phi)
	Transfer RNA genes	trnA-UGC ^ab^, trnC-GCA, trnD-GUC, trnE-UUC, trnF-GAA, trnG-GCC(ses), trnH-GUG, trnI-CAU ^a^, trnI-GAU ^ab^, trnK-UUU ^b^, trnL-CAA ^a^, trnL-UAA ^b^, trnL-UAG, trnM-CAU(ses), trnM-CAU ^a^(phi), trnN-GUU ^a^, trnP-UGG, trnQ-UUG, trnR-ACG ^a^, trnR-UCU, trnS-CGA ^b^(ses), trnS-GCU, trnS-GGA, trnS-UGA, trnT-GGU, trnT-UGU, trnV-GAC ^a^, trnV-UAC ^b^, trnW-CCA, trnY-GUA, trnfM-CAU(ses)
Genes for photosynthesis	Subunits of ATP synthase	atpA, atpB, atpE, atpF ^b^, atpH, atpI
	Subunits of NADH dehydrogenase	ndhA ^b^(ses), ndhA(phi), ndhB ^ab^, ndhC, ndhD, ndhE, ndhF, ndhG, ndhH, ndhI, ndhJ, ndhK
	Subunits of photosystem I	psaA, psaB, psaC, psaI, psaJ
	Subunits of photosystem II	psbA, psbB, psbC, psbD, psbE, psbF, psbH, psbI, psbJ, psbK, psbL, psbM, psbN, psbT, psbZ
	Subunits of cytochrome	petA, petB ^b^, petD ^b^, petG, petL, petN
	Large subunit of Rubisco	rbcL
Other genes	Subunit of acetyl-CoA-carboxylase	accD
	C-type cytochrome synthesis gene	ccsA
	Envelop membrane protein	cemA
	ATP-dependent protease subunit p gene	clpP ^c^
	Maturase	matK
	Translation initiation factor	infA
Genes of unknown function	Conserved open reading frames	ycf1 ^d^(ses), ycf15 ^ad^(ses), ycf1, ycf2 ^a^, ycf3 ^c^, ycf4

^a^ Two gene copies in IRs. ^b^ Gene containing a single intron. ^c^ Gene containing two introns. ^d^ Pseudogene. ^e^ Gene divided into two independent transcription units. Ses—Genes that are particular to *A. sessilis*. phi—Genes that are particular to *A. philoxeroides.*

**Table 3:** Genes containing introns in the *A. sessilis* chloroplast genome.

Gene	Location	Intron Number	Exon I	Intron I	Exon II	Intron II	Exon III	CDS Length
*trnK-UUU*	LSC	1	37	2538	35			72
*rps16*	LSC	1	42	911	213			255
*trnS-CGA*	LSC	1	31	713	60			91
*atpF*	LSC	1	148	800	410			558
*rpoC1*	LSC	1	435	771	1602			2037
*ycf3*	LSC	2	124	764	230	774	153	507
*trnL-UAA*	LSC	1	35	650	50			85
*trnV-UAC*	LSC	1	38	595	35			73
*rps12*	IRa		114	-	232	538	26	372
*clpP*	LSC	2	69	600	293	875	226	588
*petB*	LSC	1	6	769	642			648
*petD*	LSC	1	8	774	475			483
*rpl16*	LSC	1	9	1055	402			411
*ndhB*	IRb	1	778	667	758			1536
*rps12*	IRb		232	-	26	538	114	372
*trnI-GAU*	IRb	1	37	946	35			72
*trnA-UGC*	IRb	1	38	825	36			74
*ndhA*	SSC	1	556	959	539			1095
*trnA-UGC*	IRa	1	38	825	36			74
*trnI-GAU*	IRa	1	37	946	35			72
*ndhB*	IRa	1	778	667	758			1536

## Data Availability

The complete chloroplast genome sequence of *Alternanthera sessilis* is publicly available online in the NCBI GenBank with the specific accession number PP239384.

## Supplementary Materials

The following supporting information can be downloaded at https://www.mdpi.com/article/10.3390/genes15050544/s1, Table S1: Relative synonymous codon usage of cp genomes in *A. sessilis* and *A. philoxeroides*; Table S2: Synonymous and non-synonymous substitution rates and Ka/Ks ratio of 55 protein-coding genes in *A. sessilis* and *A. philoxeroides*.
